# Navigating an enigma: the continuing journey of autoimmunity discoveries

**DOI:** 10.1172/JCI182287

**Published:** 2024-06-03

**Authors:** Mariana J. Kaplan

**Affiliations:** National Institute of Arthritis and Musculoskeletal and Skin Diseases, NIH, Bethesda, Maryland, USA.

Autoimmune diseases (ADs) constitute about one hundred and fifty conditions that often afflict people in the prime of their life. ADs are strikingly more common in women and affect around 9% of the US population (1). Seminal work performed in the first half of the twentieth century by scientific giants such as Macfarlane, Medawar, Rose, and many others defined key concepts such as immune tolerance and immune responses to self-antigens. The last half-century has witnessed transformative breakthroughs toward understanding the pathogenesis and clinical features of ADs.

The identification in the mid-to-late 20th century of autoantibodies, which target the body’s own tissues, and their association in some cases with specific ADs, their severity, prognosis, and risk of exacerbation were critical breakthroughs in understanding systemic lupus erythematosus (SLE) (2) and other ADs. Individuals who go on to develop certain ADs characteristically synthesize autoantibodies that target intracellular and extracellular autoantigens; these autoantibodies can be detected many years before the onset of clinical disease (3). The description of antinuclear antibodies and their links to SLE and other systemic rheumatic diseases and the discovery of anticitrullinated protein antibodies in rheumatoid arthritis (RA) revolutionized diagnostic approaches and understanding of disease pathogenesis (4, 5). Substantial progress over the last several decades has helped identify mechanisms of enhanced/modified autoantigen generation in predisposed hosts that may promote aberrant immune responses to these autoantigens (6) as well as to the formation of immune complexes (7).

Increased understanding of how cell death pathways or impaired clearance of dead cells (6, 8) contribute to autoantigen generation and persistence illuminated crucial initial events in aberrant immune responses. Subsequent work revealed how modifications of self-proteins and DNA triggered by environmental stimuli may contribute to breaking tolerance in many ADs (4, 9). In addition, characterizing the intracellular pathways that sense “self” and “non-self” nucleic acids and other danger signals has transformed our understanding of mechanisms that can contribute to loss of tolerance. Among them are the endosomal TLR pathways that can sense endogenous DNA and RNA; the cGAS/STING pathway that responds to cytosolic DNA; the RIG-I/MAVS pathway, which responds to RNAs; and various inflammasome pathways that can sense several stress-associated stimuli. Indeed, dysregulation in these pathways has been associated with chronic inflammation and autoimmunity (10).

Genetic and epigenetic factors play key roles in AD predisposition, severity, and prognosis. A cornerstone in understanding how genes promote disease susceptibility was the identification of HLA-specific alleles that are linked to various ADs, such as HLA-B27 in ankylosing spondylitis (11, 12) and the shared epitope HLA-DRB1 in RA (13) among others. Significant advances in genetic technology, particularly the development of high-throughput sequencing, facilitated large-scale GWAS, which promoted the identification of non-HLA genetic loci associated with many ADs, contributing to the promotion of a better understanding of the genetic architecture underlying autoimmunity (14). Some examples include the identification of *STAT4*, *PTPN22*, *TYK2*, *CD40*, and *TNFAIP3* as candidate genes for various systemic ADs such as RA and/or SLE (15). Furthermore, the application of whole-exome and whole-genome sequencing in characterizing monogenic alterations that lead to ADs has enhanced understanding of the pathways that become dysregulated in several of these conditions. An example is the characterization of *TLR7* gain-of-function genetic variations causing SLE (16). Furthermore, somatic mutations in hematological precursor cells have been recently described to cause adult-onset, severe inflammatory rheumatic diseases (17).

Discoveries on the role of epigenetics in ADs have contributed to unraveling the complex interplay between genetic predisposition and environmental triggers. Epigenetic modifications, including DNA methylation, nucleic acid oxidation, histone acetylation, and microRNA expression, have been shown to play fundamental roles in regulating gene expression and immune responses in ADs (9). Examples in SLE include the association of T cell hypomethylation with acquisition of an autoreactive phenotype (18) as well as abnormalities in trained immunity and myelopoiesis with downstream pathogenic consequences (19). These observations have provided insights into the mechanisms underlying immune dysregulation and the possibility of targeted interventions to reverse or modulate these epigenetic changes.

The characterization of the role of sex chromosome and sex hormones (20, 21), immune-senescence, and metabolic dysfunction (including mitochondrial dysregulation) have contributed to further advancing the understanding of putative pathways of immune dysregulation that have potential therapeutic implications in ADs (22, 23).

## Immunological treatments for ADs

Substantial advances in T cell biology, including the characterization of Tregs and autoreactive lymphocytes and better understanding of the B and T cell subsets that regulate immune tolerance, marked a paradigm shift in autoimmunity research. Indeed, characterizing the role of regulatory cells has provided insights into immune homeostasis and how their dysfunction contributes to ADs. In addition, the discovery of specific cytokine pathways and their role in AD pathogenesis fueled the development of targeted therapies and biologics that have revolutionized treatment approaches. For example, TNF-α inhibitors revolutionized the management of RA, psoriasis, and inflammatory bowel disease (24). The identification of interleukins and their receptors as fundamental mediators of inflammation in ADs led to the development of biologics targeting the IL-6 receptor, the type I interferon receptor, IL-17, and IL-23, which have proven to be efficient in treating various seronegative spondyloarthropathies, other arthritides, and SLE (25). Furthermore, the identification of the JAK/STAT pathway, a fundamental signaling cascade by which cytokines and other molecules act in various immune and stromal cells, led to the development of inhibitors that can modulate inflammation triggered by multiple soluble factors, with therapeutic implications in RA, SLE, and various ADs affecting the skin and other organs (26).

Understanding the role that both innate and adaptive dysregulated immune responses play in driving and perpetuating disease has led to a much better characterization of the initial mechanisms that break tolerance in many ADs. Indeed, implementing combined therapeutic approaches that target specific innate and adaptive aspects of immune dysregulation while sparing components of the immune system that are crucial in host defense is a goal that could revolutionize treatment strategies in ADs. In addition, progress in understanding and treating other immune system–related diseases, including COVID-19 and cancer, has profound implications for treating autoimmunity. Recently, the use of chimeric antigen receptor T cells (CAR T cells) has been expanded from cancer treatment to successfully treat autoimmunity preclinically and clinically in small cohorts of patients with SLE, idiopathic inflammatory myopathies, and progressive systemic sclerosis, an exciting finding that holds promise in expanding the therapeutic armamentarium in patients with a variety of organ-specific and systemic ADs (27).

## Microbial influences in autoimmunity

The exploration of microbial insults as well as the microbiome’s role in AD has added a new dimension to understanding disease development and perpetuation. Advances in sequencing technologies have contributed to the characterization of the microbiota in various ADs, revealing alterations in microbial composition and diversity. In particular, the gut microbiome and the development of dysbiosis have been implicated in shaping immune responses and influencing the risk of ADs, such as inflammatory bowel disease, type I diabetes, multiple sclerosis, SLE, and RA (28). Certain viral infections, including EBV, have been associated with risk for multiple sclerosis, RA, and SLE (29). This understanding of the interplay between the microbiome and immune responses is fostering research into putative therapeutic interventions targeting dysbiosis or dysregulated responses to viruses and other microbes.

## Future directions for the field

The development of groundbreaking technologies is increasing the promise of targeted therapeutic approaches. For example, gene editing technologies, such as CRISPR/Cas9, may allow for immune system manipulation at a genetic level. Furthermore, using artificial intelligence in autoimmunity research offers wonderful opportunities for data analysis, predictive modeling, and pattern recognition, including identifying complex relationships in genetic and clinical data and developing predictive tools for disease risk in real-time situations.

As these breakthroughs are celebrated, we must grapple with the many persisting challenges and unanswered questions that continue to shape the landscape of autoimmune research. One key hurdle is the vast clinical and molecular heterogeneity in ADs, which clearly hampers the implementation and efficacy of various therapies. The promise that using personalized medicine approaches has in ADs is tempered by these complexities in immune responses. In addition, despite all these advances, the exact triggers of ADs remain elusive, and fully explaining how infections and other evolving environmental factors contribute to disease onset and disease severity remains a substantial challenge. The dynamic nature of the microbiome in each person throughout their lifetime and upon exogenous and endogenous triggers also requires further study. We also need to consider why autoimmunity affects women in disproportionate numbers. Several attractive hypotheses have been proposed, but the detailed mechanisms remain to be better characterized.

Progress in all these areas is needed to realize the hope that, one day, we might be able to prevent the development of ADs. In that regard, the identification of reliable biomarkers of disease risk, before irreversible damage has occurred, remains a phenomenal challenge. Finally, tailoring therapies based on genetic and epigenetic makeup and immune dysregulation profile presents many hurdles, including persistent health disparities, that must be very carefully addressed to achieve full success in treating ADs.

The last half century has yielded fantastic progress in our understanding of ADs, which have not only transformed diagnostic and therapeutic approaches but have also paved the way for precision medicine in these conditions. This epic journey from the identification of autoantibodies to manipulating the microbiome stems from the collaborative efforts of many researchers, underscoring the interdisciplinary nature of autoimmunity research, and provides hope for a future in which ADs will be better defined, understood, managed, and even prevented. Collaborative efforts, interdisciplinary approaches, and rapidly evolving technologies will hopefully contribute to answering lingering questions and ushering in a new era of personalized, effective, and widely available therapeutic strategies in ADs.

## Version 1 06/03/2024

Electronic publication
